# Entering a liminal state when becoming a social prescribing link worker and how it affects retention: findings from a UK qualitative study

**DOI:** 10.1017/S1463423625100534

**Published:** 2025-11-11

**Authors:** Stephanie Tierney, Lucy Moore, Debra Westlake, Shoba Dawson, Emma Fuller, Kerryn Husk, David Nunan, Pauline Roberts, Lilly Sabir, Jane R. Smith, Obioha C. Ukoumunne, Kamal R. Mahtani

**Affiliations:** 1 Nuffield Department of Primary Care Health Sciences, University of Oxford, Oxford, UK; 2 School of Medicine and Population Health, University of Sheffield, Sheffield, UK; 3 Oxford City Primary Care Services, Oxford, UK; 4 Peninsula Medical School, University of Plymouth, Plymouth, UK; 5 Oxford Health NHS Foundation Trust, Oxford, UK; 6 The Centre for Psychological Research, Oxford Brookes University, Oxford, UK; 7 Department of Health and Community Sciences, University of Exeter, Exeter, UK

**Keywords:** Primary care, qualitative research, social prescribing, staff retention, workforce

## Abstract

**Aim:**

To explore factors shaping social prescribing (SP) link workers (LWs) experiences of their job, and how they influence decisions about whether or not to leave it.

**Background:**

LWs support healthcare delivery by listening to patients’ non-medical concerns and social or relational difficulties, connecting them to ‘community assets’ (groups, organizations, charities) when relevant to help. LWs try to assist people with often complex emotional and/or social issues. This can affect how they feel in their job.

**Methods:**

As part of a mixed methods project on LW retention, a qualitative study was conducted. It involved 20 LWs, purposively selected from respondents to a questionnaire; variation in the sample was sought in terms of self-efficacy in the role, length of time in it, intention to leave or not, employing organization, where they worked in the UK and gender. Semi-structured interviews, conducted via Microsoft Teams, were audio-recorded and transcribed verbatim. Prior to interviews, we asked participants to take photographs of: a typical part of their working day; something that gave them confidence in their role; an unexpected part of their role. These photographs were discussed at the start of the interview. Thematic analysis was used to interpret data (the computer programme NVIVO supported this); this involved coding and clustering codes to develop analytical themes.

**Findings:**

We produced four themes from the data; 1) Disconnection through place and space: straddling different organizational spheres; 2) Delivery ambiguity: vagueness around the link worker role; 3) Job misalignment and realignment: navigating identity and boundaries; 4) Clouded by instability: uncertainty around career advancement and sustainability. This led to the development of an overarching theme of LWs inhabiting a liminal space as they entered and undertook the role. Findings highlight the importance of training, supervision and other support to ensure LWs do not experience a prolonged liminal state.

## Introduction

### Social prescribing and the link worker role

In recent years, social prescribing (SP) has gained increasing global traction (Morse et al., 2022; Muhl et al., 2023) as a means of addressing non-medical issues (e.g. loneliness, housing or financial problems) affecting people’s health and well-being (Buck and Ewbank, 2020). It involves connecting people to non-medical support (e.g. groups, charities, organizations) often in the voluntary-community-social enterprise (VCSE) sector (NHS England, 2020) (e.g. gardening groups, befriending services, housing advice, food banks, book clubs, art classes, volunteering opportunities).

In the United Kingdom (UK), this connecting role is undertaken by someone called a link worker (LW) or social prescriber. SP has been rolled out at scale across the UK. However, there are some differences in implementation, context and delivery across devolved nations. In England, the dominant (funded) model is through primary care, with referrals via general practice to a LW employed through primary care or sub-contracted to a VCSE organization (Husk and Sanderson, 2024; Tierney et al., 2025a). In Scotland, most LWs are based outside of the health service and are community-based, with the Scottish government actively targeting SP delivery to deprived areas of the country (McSwiggan et al., 2025). In Wales, there is a more diffuse approach to SP; it supports multiple and varied points of access (e.g. through self-referral, community). This is supported by LWs who are based in mostly Local Authorities and community settings, emphasizing the health promotion, prevention and early intervention aspects that SP might offer (Wallace et al., 2021).

### Link worker burnout and retention

Previous UK research has highlighted that burnout experienced by LWs prompts them to consider their career options and longevity in the job (Fixsen et al., 2022; Westlake et al., 2024). A survey by the National Association of Link Workers (2020) found that one in three LWs taking part, from England, were considering leaving their post. A more recent questionnaire we distributed, as part of a mixed methods study (Tierney et al., 2025b), found that one in two LWs who responded, from across the UK, had thought about resigning from their job in the previous six months.

Loss of LWs jeopardizes the sustainability of SP in primary care. These individuals bring with them or develop rich tacit knowledge of local community assets. A large part of a LW’s role is developing relationships with staff in primary care and in the VCSE. When a LW leaves their post, they take with them a wealth of expertise and connections that may not be easily substituted by their replacement, who will need time to develop their own networks.

To help prevent workforce turnover, research is needed to understand how LWs experience their role and develop a professional identity. Professional identity relates to how individuals perceive themselves and their occupation in terms of attitudes, values, knowledge and skills (Adams et al., 2006). It involves learning to be part of a professional group (Poole and Patterson, 2021). For healthcare practitioners, professional identity can be engendered through education (Matthews et al., 2019; Poole and Patterson, 2021) and role modelling (Koh et al., 2023). For LWs, such avenues are missing as they do not have to undertake a qualification in SP. They might also be the only LW based in a primary care setting.

We have explored, as part of a mixed methods study, the impact of occupational self-efficacy and job discrepancy on the intention of LWs based in the UK to leave their role. Occupational self-efficacy relates to Bandura’s (1986) social cognitive theory. Self-efficacy reflects the beliefs someone has around their ability to act in a situation (Bandura, 2006). Bandura argued that people could achieve goals, even when facing challenges, if they believed they had the capability to execute actions required of them (Bandura, 1997). Higher self-efficacy has been reported as protective against stress and burnout in the workplace (Fida et al., 2018), and has been associated with people more positively assessing their job (Bandura, 1997; Laschinger et al., 2015). Job discrepancy relates to the gap between expectations and reality; our previous research (Tierney et al., 2025b), and that of others (Rhodes and Bell, 2021), has highlighted a potential discrepancy between job descriptions and what being a LW is actually like.

For the first part of our mixed methods study, we received 342 useable responses to an online questionnaire sent to LWs across the UK. It included sections on occupational self-efficacy and job discrepancy. Results are reported elsewhere (Tierney et al., 2025b). This was followed by qualitative research, findings from which we present below.

## Methods

This qualitative study set out to address the question: What are LWs experiences of the role, and how does this shape their view on staying in or leaving it? Ethics approval was provided by the University of Oxford (R87383/RE001).

### Recruitment and participants

When completing the questionnaire that formed the first part of the mixed methods study (Tierney et al., 2025b), LWs were asked if they were willing to be contacted for a follow-up interview; more than half agreed to this. Recruitment emails were sent out to selected LWs during January–February 2024. A participant information sheet was attached explaining what was involved if they took part in an interview, including data protection and confidentiality procedures.

Purposive sampling was used. Initially, we selected individuals who varied in self-efficacy scores, how long they had been in their post, and whether they had considered leaving it or not. As recruitment continued, we sought further variation in terms of job discrepancy scores, employing organization (VCSE, primary care, council), where they worked within the UK and gender.

At the outset, based on previous research and the idea of information power (Malterud et al., 2016), we felt that up to 20 interviews would be sufficient to provide a depth of insight required to develop analytical themes. However, we were prepared to continue recruiting if we felt that our understanding of key concepts needed refining. As we had regular meetings about the data, as data collection progressed, we were able to identify that after 18 interviews we had a good sense of key concepts developed from the data. We conducted another couple of interviews but this did not raise new ideas. Therefore, we stopped recruiting. Everyone we approached agreed to be interviewed; this was not surprising given that they had shown their interest in taking part through the questionnaire.

### Data collection

Data collection consisted of an online, one-to-one semi-structured interview that was audio-recorded, with the LW’s consent. Prior to the interview, LWs were asked to take three photographs (see Table 1 for details). In line with photo elicitation (Harper, 2002; Glaw et al., 2017), photographs were discussed at the start of the interview. The researcher commenced the interview by asking the LW to describe the photographs they had taken and why. This allowed the interviewee to have some control of topics covered, ensuring that from the outset they could raise issues that were most relevant for them (Cassell et al., 2020). Interviews were conversational in nature and supported by a topic guide (see Supplementary file 1). Interviews were conducted by one of the authors (LM or ST) between January–April 2024. They lasted between 30–80 minutes.

**Table 1.** Topics participants were asked to take photographs on

Photo 1	One that showed a typical part of their working day
Photo 2	One that showed something that gave them confidence in their role
Photo 3	One that reflected an unexpected part of their role or something they did on a regular basis that was not in their job description

N.B. Participants were advised not to take photos of people or identifiable data. All used their mobile phones (disposable cameras were available if necessary) and emailed photos to a researcher prior to the interview.

### Data analysis

Thematic analysis, as outlined by Braun and Clarke (2022), was used. The initial stage, conducted by LM, included listening to the audio-recordings, cross-checking for accuracy and reading transcripts to become familiar with the dataset. Following this stage, an early coding structure was developed by LM, with brief descriptions assigned to each code, identifying how LWs described their experiences in the role. These were discussed and refined through coding conversations between LM and ST, and then shared at meetings with the rest of the research team. This culminated in the development of a mind-map of main concepts (see Supplementary file 2), which helped us to develop the final themes. NVivo 14 software was used to organize data and to record early memos and annotations.

As findings developed, we shared our thinking with a patient and public involvement (PPI) group. They helped us to refine our analysis. This group, who we met with on three occasions, consisted of six lay people with an interest in SP as part of holistic patient care. We discussed with this group tensions for LWs in terms of employment and management structures, location and allocation of work and how this affected professional identity in the role.

## Findings

Our purposive sampling resulted in five LWs from North West England, four from South West England, three from South East England, two from London, two from East of England, one from North East England, two from Scotland and one from Wales. Characteristics of these interviewees are listed in Table 2. A gallery of photos they produced can be found on our study webpage. Interviewees stated that taking the photographs was helpful in getting them to think about their role in advance of the interview. The photographs highlighted to the research team some of the unexpected issues LWs dealt with (e.g. rehousing pets) and the difficulties they encountered when working remotely or when faced with mounting record keeping. We developed, from the data, four themes, which we now outline.

**Table 2.** Characteristics of LWs taking part in an interview

Study identity	Gender	Age range	Time in role	Employer	Thinking of leaving job at time of interview
**LW01**	Female	21–30	21–24 months	Primary care	Yes
**LW02**	Female	41–50	>3 years	Primary care	Yes
**LW03**	Female	21–30	13–16 months	Primary care	No
**LW04**	Female	31–40	>3 years	Primary care	Yes
**LW05**	Female	41–50	13–16 months	Primary care	Unsure
**LW06**	Male	51–60	7–12 months	Primary care	Yes
**LW07**	Female	51–60	17–20 months	Primary care	Unsure
**LW08**	Female	21–30	1–3 months	VCSE	No
**LW09**	Female	51–60	>3 years	Primary care	Yes
**LW10**	Male	>60	13–16 months	Primary care	No
**LW11**	Male	41–50	7–12 months	VCSE	Unsure
**LW12**	Female	31–40	33–36 months	VCSE	Yes
**LW13**	Female	31–40	>3 years	Council	Yes
**LW14**	Female	41–50	21–24 months	VCSE	Unsure
**LW15**	Female	21–30	4–6 months	VCSE	No
**LW16**	Female	31–40	17–20 months	Primary care	Yes
**LW17**	Female	31–40	>3 years	VCSE	Unsure
**LW18**	Female	41–50	>3 years	VCSE	Unsure
**LW19**	Female	51–60	7–12 months	Council	No
**LW20**	Female	41–50	>3 years	VCSE	Unsure

**Theme 1: Disconnection through place and space: straddling different organizational spheres**

*‘…although we work in both settings (charity and primary care) it almost feels like we’re kind of just teleporting in and out of GP practices. It feels like we’re not really present in there if that makes sense.’ (LW08)*

This theme reflects the position LWs described occupying between health and the VCSE sector, with some stating they felt unwelcome in these settings. Spanning more than one setting could impede their sense of having a clear professional identity because they felt a lack of affiliation, especially to primary care. They also described the difficulty of experiencing variation in working styles across organizations, which they had to accommodate:

*‘I’m employed by the (name of charity) and funded by the NHS…The difference between the charity versus the NHS sector…it’s just a different pace. I think the charity that I’m in…is quite chill and then the NHS is a lot more busier and sort of stressful.’ (LW01)*

Those employed through the VCSE sector were more likely to mention flexibility in the role, and being able to develop and try new initiatives and ways of working; this helped create a sense of professional identity and contributed to a wish to stay in the role:

*‘I love the variety…So actually I probably see more longevity in this role…I’m not sat behind a desk all the time.’ (LW14)*

However, the nature of LWs’ employment, spanning the VCSE sector and health, could lead to feelings of uncertainty, especially when answerable to more than one manager; LWs employed through non primary care routes (e.g. VCSE), in particular, would question who to approach or who had the final say over what they did:

*‘I’ve got two bosses basically! [laughs] Well, I’ve got three bosses. So I’ve got my boss at my company, I’ve got to keep the practice manager and the doctors here happy, cause they’re also my bosses, and then we’ve got the PCN [Primary Care Network] lead…we’ve also got to keep him happy…Keeping them all happy sometimes is a lot.’ (LW11)*

Data highlighted that a silo mentality could occur when LWs were dislocated from primary care staff. This happened if LWs were not invited to team meetings or did not have a good induction. Such silo working led to a potential division – an ‘us and them’ mentality:

*‘…I don’t necessarily feel part of the GP surgeries. Although I’ve been going for four years, and referrals are full…it sort of is, like I go in, I sit in my little room by myself all day, seeing people. Then I go home…. at the beginning I did presentations and stuff like that and there is people I can message and ask questions but it does feel a little bit isolating.’ (LW13)*

Workspace in GP practices affected LWs’ sense of identity and dislocation. When rooms were unavailable, LWs were home-based – gaining flexibility and autonomy but potentially feeling isolated and not part of an organization. Spaces allocated to LWs in primary care for seeing patients could be transient. An office in a GP practice was often depersonalized because it was a shared space with clinical staff. When LWs had scope to personalize their room, it made them feel that where they worked connected with their purpose and aims for the job:

*‘It’s really to just make it feel less clinical. [In the room] we have tissues because we get lots of upset patients…and sort of ideas really that we can point to for things that we signpost to frequently…I feel ready for the patients to come in and share their stories. I feel like it’s an inviting, relaxed space for them.’ (LW03)*

Some interviewees mentioned working in rooms lacking daylight (LW07; LW10), which were described as a box (LW11) or broom cupboard (LW19). Presence of clinical equipment, such as a blood pressure machine (LW19) or examination lamps on the wall (LW07), were a visual indicator that ran counter to LWs’ social, holistic role. LW14 was told to see patients in the surgery’s waiting room; this idea was rejected as it was an inappropriate space, lacking privacy and not somewhere the LW could have meaningful discussions with patients. It suggested a lack of understanding by others of what LWs did. This contributed to the transitionary state LWs encountered, as discussed in the next theme.

**Theme 2: Delivery ambiguity: vagueness around the link worker role**

‘*The receptionists don’t know. Practice manager doesn’t know. GPs don’t know and my manager has never looked at any of our work…I think it’s just an example of how there’s lack of organisation. Lack of input maybe.’ (LW13)*

This theme highlights indistinctiveness around the LW role, leading to misunderstandings about its scope and nature. This relates to how others perceived the role (and consequences of this), which interviewees stated was less defined than others in primary care (e.g. in NHS documents). This could lead to inappropriate referrals (e.g. from GPs) or having the support they provided wrongly described to patients:

*‘I think the phrase that we use is that social prescribing is the dustbin [laughs]… anything that the doctors can’t deal with in 20 minutes or feels is inappropriate to deal with goes to us and we’re expected to cope with it.’ (LW07)*

*‘…it’s a very common thing for us that we get referrals for more complex needs than we’re meant to…a lot of the time the GPs will say that we’re counsellors when we’re not which I then have to, it’s first session going we’re not counsellors.’ (LW08)*

Such misunderstandings could stem from LWs’ professional status, which some interviewees depicted as compromised by lack of a specific SP qualification; this made them concerned about their credibility within medical settings:

*‘…in the NHS if you’re a doctor or you’re a nurse, you have that qualification and you follow that pathway…if they want to embed us in GP practices…I think a formal qualification should be gained…I think they look at us as ‘you’re unqualified, anyone can do it’ sort of thing.’ (LW05)*

Having good managers was considered essential in helping people to settle into and feel at ease with their job as a LW. This called for a clear understanding of the purpose of SP. Misunderstandings in relation to the role could mean that LWs failed to receive the support/supervision required. This was unfortunate as good supervision seemed to be important in helping people achieve a good work-life balance. This was not always the experience of interviewees; some talked about the real impact that undertaking this role had on their own well-being, which promoted them to consider leaving:

*‘…sometimes you want to go home and switch off and sometimes I can’t do that in this job and that’s emotionally draining…’ (LW01)*

*‘My friends go to work, they do their job and they come home, they don’t have, I can leave here feeling sometimes a bit glum…I feel a bit stressed…’ (LW02)*

Conversely, those wanting to stay in the role described a work environment in which self-care was actively encouraged:

*‘…one of the things they (managers) really bang into us is you do not take that work home for the weekend. If there’s anything bubbling up on the surface I don’t have just one manager, I can go to the other managers within the team because they don’t want us to take that home.’ (LW19)*

This relates to the idea of job expectations, which forms part of the next theme.

**Theme 3: Job misalignment and realignment: navigating identity and boundaries**

*‘…if you’re coming in…thinking you’re doing a job that is all community-based and getting people to the right place, and then you’re finding it’s…sitting here, seeing patient after patient after patient, then you might think what am I doing?’ (LW09)*

This theme relates to the changing remit of the role, and how this aligned or not with LWs’ personal views of it. Several interviewees described coming to the post believing there was a good fit between their experience and knowledge (e.g. after having worked in the VCSE sector or having developed communication skills or having undertaken mental health training). However, reality often differed from their expectations. It was noted that the scope of the job could become more or less varied over time, leaving LWs destabilized or frustrated:

*‘…the Household Support Fund has become a huge part of my work. When I first started…there was a big mix. It was things like support groups for dementia, it was things like social isolation…It’s shifted probably about three or four months ago…and it’s all money. Everybody I see has got problems with money, either debts or it’s just general cost of living more than debts…’ (LW11)*

*‘…when the team first started, it was very, very focussed on isolation and loneliness, specifically in older people so it was a very different job to how it is now…now it is, homelessness, addiction, violence, domestic violence, discharge from prison. Severe and enduring mental health. The list just goes on…’ (LW13)*

The shifting scope of duties was a key issue when COVID-19 was rife; for most interviewees, this fluidity continued post-pandemic. Several interviewees mentioned being unable to practice as expected because the medical model dominated how SP was delivered; tensions were experienced around ways of working for a role that aimed to address social factors that was situated within a medical environment. This led to pressure to spend less time with patients or developing community connections, which misaligned with LWs’ view of their job:

*‘According to the NHS framework, social prescribing is…decreasing social isolation, it’s increasing community involvement, it’s getting out and organising community projects…I haven’t got time for that…There’s no way that the surgery would let me do that…I’m really experienced with volunteers…I have that skill set, but there’s no way that would happen.’ (LW11)*

*‘…this one particular practice…the Business Manager contacted me, I was a little bit surprised that they were coming at me with quite fixed ideas about what social prescribing is and what it shouldn’t be, so I had to have quite a candid conversation…We had to talk about how it’s not the medical model but more leaning into the social model of disability…’ (LW19)*

A disconnect between LW expectations and reality was compounded by inaccurate job descriptions that overlooked the variety and complexity of the role, a comment made by several interviewees. Some also mentioned not receiving guidance around their role when they started; about being left to craft it for themselves. Connecting with peers could help with clarifying job-related boundaries. It also helped LWs to appreciate what made their role unique:

*‘… it sounds like I’ve got the perfect team here, but they really are really good, honestly, if we’re in a team meeting there is no fear of saying this isn’t going to work or I’m worried about this, you know, and I think that’s made a difference, I think that’s helped…’ (LW18)*

When such support was missing, individuals described going out and finding it for themselves (e.g. from coaches or mentors), to make the role tolerable and to cope with the instability associated with it. This is the focus of the final theme.

**Theme 4: Clouded by instability: uncertainty around career advancement and sustainability**

*‘…it’s tricky because we’re up for funding at the moment so I actually don’t know if I’ve even got a job come the end of March…’ (LW01)*

This theme highlights how working conditions encountered by LWs, in terms of job security and progression, left them feeling they occupied a transient work position requiring versatility and an ability to tolerate change and ambiguity:

*‘…it’s unstable contracts…a lot of people on our team have been disbursed throughout our charity…so it’s very tempting to kind of take a more stable role within the NHS within mental healthcare where I’m not fighting for funding constantly, where it’s not that constant, is it going to be here in a year?’ (LW08)*

Lack of career progression was a key factor that prompted people to consider leaving their role, as suggested in the following data extracts:

*‘…If you’re not developing and if you’re not progressing then you need to leave, you need to give yourself that push.’ (LW17)*

*‘…I don’t think I can go anywhere with this role. I can’t see a higher level where I am…’ (LW02)*

The last of these interviewees noted that being in the role could open doors to other opportunities, through working closely with voluntary organizations and charities. Others noted that being employed through the NHS offered access to more career options, in contrast to being employed in the VCSE sector:

*‘The NHS is broad, if that social prescriber wants to do a sideways move, they can…if they want to do an upwards move they can.’ (LW11)*

*‘…because it’s a small charity…if there were roles to progress into, it would be outside…not within my charity.’ (LW15)*

Some individuals did not want to take on more responsibility, meaning they were happy to stay in their role. Others said that because they did not develop skills or experience in the role, it was hard when applying for a new post, leading them to consider remaining as a LW:

*‘I think our jobs are very niche, so when you look for another job…I’ve had knockbacks…and I think it’s because our job is very niche.’ (LW05)*

A few interviewees talked about salary and career advancement not being a concern; this seemed to be the case if older and coming to the role later in life. In contrast, several interviewees said they were not remunerated for the level of risk their work entailed. However, discomfort was expressed when raising the issue of money in relation to a job associated with gaining satisfaction from helping others:

*‘…I don’t want to be motivated by money, I really, really don’t, because that’s not why I do this job but it does have a bit of an impact. I’ve just built a big house, me and my husband, and it’s cost a lot more than what we thought and that’s had a bit of a financial strain on us…’(LW02)*

Some LWs suggested that changes in their work environment made them feel more uncertain (e.g. a new manager meaning that supervision was less invested in) or more in control and stabilized (e.g. structures established that enabled people to settle into the role – such as induction processes or training opportunities). Those thinking about leaving their role, in particular, spoke about its insecure nature, due to things like finances and short-term contracts, receiving increasingly complex cases; these were areas over which they felt they had little control, which were important for their sense of security in the post. This could lead them to question how much difference they were able to make to an individual’s life, a key driver for their entry into and willingness to remain in the role:

*‘I think most people that go into jobs like this do it because they want to help people and that desire can override a lot of the challenges.’ (LW13)*

### Overarching theme: inhabiting a liminal state

Themes presented above show that LWs we interviewed inhabited a role that spanned primary care and the VCSE sector, which was associated with insecurity and instability. Professional identity could be impacted by a misalignment between expectations of the role and reality, caused by ambiguity around what being a LW entailed. This relates to conversations we had with the study’s PPI group, which prompted us to consider the concept of liminality as a lens through which to explore the data. For example, there was a sense among the PPI group that LWs’ descriptions of the role (based on anonymized data extracts we shared with them) suggested LWs could feel like outsiders and not fully supported. Our research highlights factors placing LWs in a liminal state, which are shown in Table 3; these included having no professional qualification specifically in this role and lacking training in it. In addition, being a new position, unfamiliar to primary care staff and patients, caused LWs to feel unstable and uncertain.

**Table 3.** Summary of factors contributing to and helping LWs to move on from liminality, and consequences of liminality for these employees

Factors contributing to a liminal state among LWs	Spanning organizations with varying ethos Poor understanding of the role Misalignment of the role with what was expected Unstable funding and poor career progression
Consequences of occupying a liminal state for LWs	Feeling undervalued Job insecurity Referrals that do not align with the LW role Job dissatisfaction Disconnection and isolation in the workplace
Factors that help LWs to move from liminal state	Access to training Supervision Clarity from managers Peer support Steps to integrate LWs into primary care (e.g. induction, meetings)

Liminality is a theoretical concept associated with anthropological studies, focused on rites of passage within different societies (van Gennep, 1961). Those experiencing liminality may encounter feelings of confusion and anxiety as they move towards a new social status (Ibarra and Obodaru, 2016; Zhao et al., 2024). Some evidence suggests there can be positive impacts associated with liminality – encouraging personal growth, a fresh perspective (Donetto et al., 2021; MacKay, 2024). However, our research suggested that for LWs, entering a liminal state upon taking up the role was a period of heightened emotions, when they could feel pushed to the edge and struggled to cope.

LWs may inhabit this liminal state for more or less time depending on the context within which they work. For example, some of those employed through a VCSE organization described the challenge of reporting to two managers (one in the VCSE organization and another in primary care), but might be more likely to encounter informal support systems (e.g. from peers). We argue that moving out of a liminal state occurred as individuals came to feel embedded in their professional identity as a LW. This transition appeared to be aided through supervision, training and peer interactions.

## Discussion

This qualitative study set out to explore factors shaping LWs’ experience of their job, and how this influenced decisions about whether to leave it. Data showed that LWs can occupy a space ‘betwixt-and-between’ (Turner, 1967) settings and organizations, leading to them feeling disconnected and like an outsider. They hold a position that can be poorly understood, without clear boundaries. Furthermore, it was described as a job lacking stability or career progression. These factors contributed to LWs embodying a liminal state. The idea of liminality has been noted as useful in helping to understand experiences in the workplace (Zhao et al., 2024), but it has not been used previously in relation to LWs. Strengths and limitations of the research are outlined in Table 4.

**Table 4.** Strengths and limitations of the research

Strengths	Limitations
A range of LWs took part in terms of locations across the UK, employment structure, time in role, career trajectory.	LWs were self-selecting, having been invited to participate in a survey at a snap-shot in time, based on their thoughts of leaving their role.
Applying the concept of liminality provided a useful framework to collate data from one-to-one interviews and allowed for an in-depth exploration of the complexity and ambiguity of LWs’ experiences in their role and their intention to quit.	Findings do not reflect the views of all LWs in the UK.

Liminality has been used to explain transitions in professional settings (Ashforth, 2000; Beech, 2011), including studies involving healthcare staff (Allan et al., 2015; Croft et al., 2015; Gordon et al., 2020). This previous work has shown that the time preceding and immediately following a role transition is a period during which identity is more ambiguous than usual (Ashforth, 2000). Our data suggested that this was the case for LWs, whose professional identity often crossed domains and whose well-being in the workplace became beholden to organizational decisions and actions.

LWs can feel as if they are ‘neither here nor there’ (Ebaugh 1988: 143), suspended betwixt-and-between (Turner, 1967) organizational routines, professional cultures and identities (Ibarra and Obodura, 2016). They may encounter a fluid liminal state whereby they mould the role to fit the situation within which they work. This may mean they are not able to fulfil work-related ambitions, as they seek to accommodate what is required by different organizations and managers. As noted by Wildman et al. (2019), they can encounter tensions between meeting high referral targets and being able to assess and address, in a holistic way, patient needs. This can be a particular issue when community support is difficult to access due to a lack of up-to-date information on what is available locally (Bertotti et al., 2018).

The liminal process starts with a triggering event (Beech, 2011) (e.g. the offer of a job as a LW). Individuals are then said to engage for a specific time in rituals (Turner, 1969) before transferring into a new state of being that is meaningful for them and their community. Entry rituals could help LWs to manage their liminal state, such as a comprehensive induction, invitations to team meetings, and having clear supervision structures that consider their well-being. LWs have to learn the ‘practical norms of professional identity’ (Zhao et al., 2024: 2) as they navigate the scope of the role, given that they do not undertake a specific professional qualification for this post. As noted above, peer support can help to foster a sense of professional identity. This relates to the writing of Turner (1967; 1969), who used the term ‘communitas’ to refer to a shared experience and sense of belonging with those in a similar liminal state. Future research on SP could identify how to develop this community of practice, although peer support should not replace more formal supervision that enables LWs to deal with the complex cases and emotional demands their job can bring (Westlake et al., 2024). Support in the role (e.g. through regular supervision and from peers) is important as LWs provide personalized care for patients, often offering emotional and motivational assistance to individuals.

It could be argued that occupying a liminal state is part of a LW’s unique boundary spanning role between healthcare and community support, which helps to foster buy-in to it (Tierney et al., 2020). Therefore, liminality may be a necessary state for these employees, requiring consideration of how to make it tolerable. Better understanding of liminality experienced by those involved in the care of patients can help to inform training and support offered (Zhao et al., 2024). This would include familiarising staff and patients with the role, ensuring appropriate referrals and realistic expectations of what LWs can do.

LWs have noted not being trained or skilled in addressing the increasingly complex cases coming before them (Wildman et al., 2019); this needs to be considered to ensure that LWs do not contemplate leaving their position. SP managers, in particular, require training when taking on this responsibility; these staff can feel unsupported in doing so (Beardmore, 2020), which is a concern given the importance placed on strong managers by our interviewees and the role they can play in helping LWs to navigate the liminal state encountered when taking up their post. This could be an area for future research, as outlined in Table 5.

**Table 5.** Areas for future research

1	Describing mechanisms associated with LWs’ career progression and how these are fostered
2	Understanding how to create communities of practice for LWs
3	Exploring how to better integrate LWs into primary care
4	Identifying optimum models of supervision and associated costs
5	Developing training for SP leads and for those managing LWs

## Supporting information

Tierney et al. supplementary material 1 Tierney et al. supplementary material

Tierney et al. supplementary material 2 Tierney et al. supplementary material

## Supplementary material

The supplementary material for this article can be found at https://doi.org/10.1017/S1463423625100534.

## Data sharing

Due to the consent process for data collection with participants, no data can be shared except for quotations in reports, journal articles and presentations.
