# Supplementary material for: Entering a liminal state when becoming a social prescribing link worker and how it affects retention: findings from a UK qualitative study
Source: Prim Health Care Res Dev. 2025 Nov 11;26:e92. doi: 10.1017/S1463423625100534 (PMC12646184; doi:10.1017/S1463423625100534)
Supplement: Tierney et al. supplementary material 1 — Tierney et al. supplementary material [file S1463423625100534sup001.docx]

**Supplementary file 1:** **Example interview questions (interviews were semi-structured so these questions should be seen as a guide to topics covered)**

| - What made you decide to become a LW? - How has your role as a LW changed since you started it? - How far has the role been what you expected it would be like? - What is the best thing for you about being a LW? - What is the most challenging thing for you being a LW? - How often, if at all, have you thought about moving on from or leaving your role as a LW? - What would you tell other people who are thinking about becoming a LW about what it is like to do the job? |
| --- |
