# Supplementary figures and images for: Entering a liminal state when becoming a social prescribing link worker and how it affects retention: findings from a UK qualitative study
Source: Prim Health Care Res Dev. 2025 Nov 11;26:e92. doi: 10.1017/S1463423625100534 (PMC12646184; doi:10.1017/S1463423625100534)

**Supplementary file 2: Mind-map of key concepts developed from the interview data**


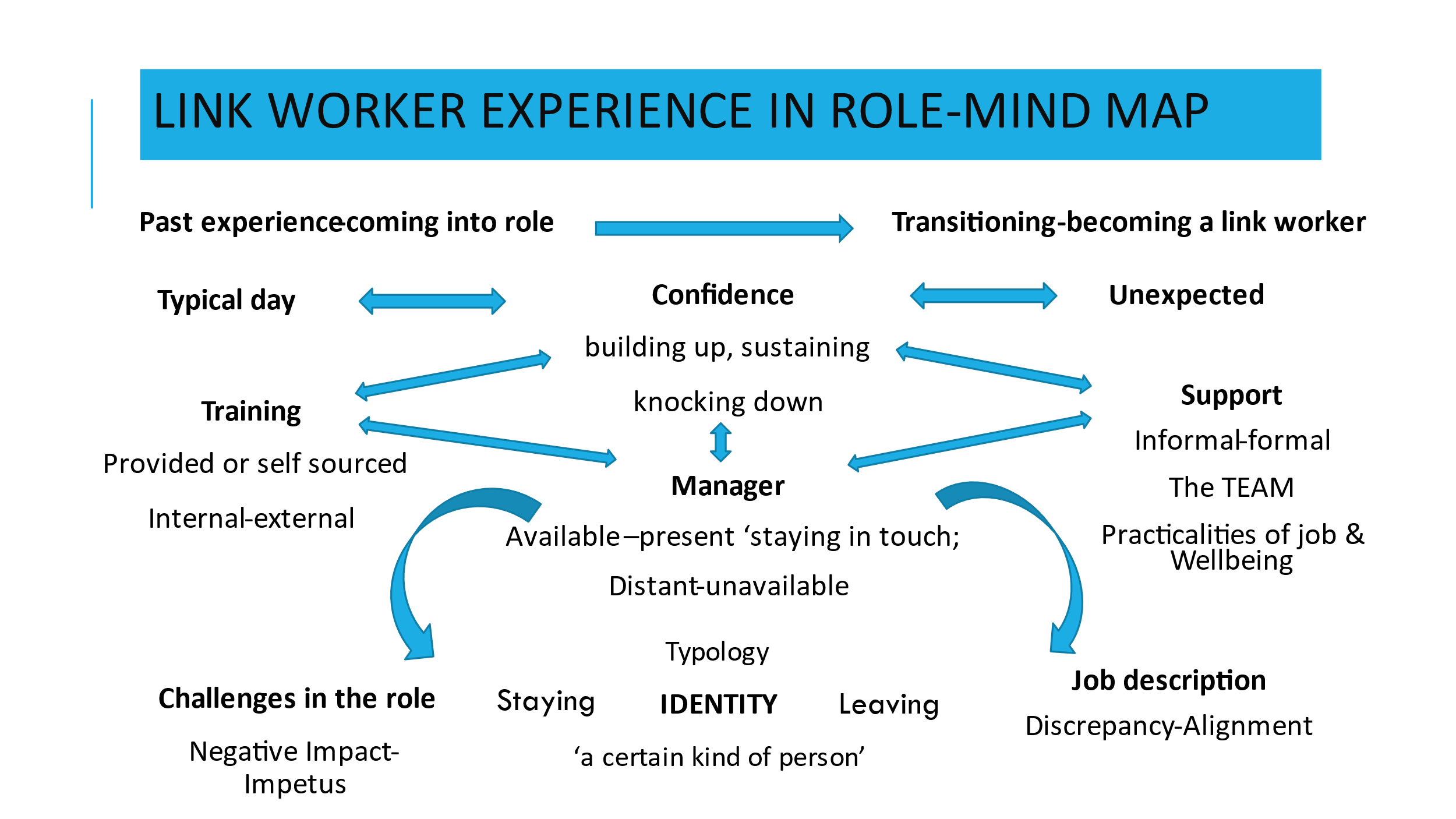

Supplement: Tierney et al. supplementary material 2 — Tierney et al. supplementary material [file S1463423625100534sup002.docx]
